# Drug-induced hyperuricemia: multi-pathway regulation, causative drugs, and individualized management strategies

**DOI:** 10.3389/fphar.2026.1791120

**Published:** 2026-06-05

**Authors:** Binfeng Xiong, Chengzheng Duan, Sheng Xu, Shiyu Xu, Chao Yang, Dongjuan He, Cheng Luo

**Affiliations:** 1 Jin hua Graduate Joint Training Base, Zhejiang Chinese Medical University, Hangzhou, China; 2 The Quzhou Affiliated Hospital of Wenzhou Medical University, Quzhou People’s Hospital, Quzhou, China; 3 Department of Nephrology, Quzhou Hospital of Traditional Chinese Medicine, Quzhou, China; 4 Department of Endocrinology, The Second People’s Hospital of Quzhou, Quzhou, China

**Keywords:** drug-induced, hyperuricemia, intervention strategy, pathogenic mechanism, uric acid metabolism

## Abstract

Hyperuricemia, a prevalent metabolic disorder, is not only the primary cause of gout but also an independent risk factor for various chronic conditions, including hypertension, cardiovascular and cerebrovascular diseases, and diabetes mellitus, thereby posing a significant threat to multi-organ health. Iatrogenic factors represent a key pathogenic trigger for hyperuricemia. With the expanding spectrum of clinical medications and the widespread adoption of polypharmacy, drug-induced hyperuricemia (DIH) now affects up to 25% of hospitalized patients and over 80% of transplant recipients on cyclosporine, emerging as a critical challenge to medication safety and therapeutic efficacy. We conducted a comprehensive literature search across PubMed, Embase, and Web of Science, systematically analyzed 76 relevant high-quality studies, and summarized the core pathogenic pathways, causative drugs, and individualized management strategies of DIH. Focusing on pharmacological mechanisms and clinical translation, this review delineates two pivotal pathogenic pathways of DIH: one involving dysregulation of key transporters that control renal uric acid reabsorption and secretion, and the other characterized by enhanced uric acid production via disruption of purine metabolism. We summarize over 10 classes of causative drugs and their molecular mechanisms, thereby advancing current understanding of DIH pathogenesis. Finally, we integrate management strategies encompassing medication adjustment, urate-lowering therapy, and non-pharmacological interventions, providing a scientific basis for rational prescribing, screening of high-risk populations, and the development of safer therapeutic agents.

## 1 Introduction

Hyperuricemia is a prevalent metabolic disease with a globally increasing incidence, evolving into a major public health issue threatening human health. Notably, this epidemiological trend directly contributes to the high incidence of related complications. Data from *The Lancet Rheumatology* show that as of 2020, the global number of gout patients has reached 55.8 million, and this figure is projected to soar to 95.8 million by 2050 (Cross et al., 2024). Accumulating evidence confirms that hyperuricemia is the direct cause of gout. The inflammatory response and tissue damage induced by monosodium urate crystal deposition constitute the core pathological basis for the development of gouty arthritis and tophi. Beyond gout, hyperuricemia serves as an independent risk factor for the occurrence and progression of multiple chronic diseases, including hypertension, cardiovascular and cerebrovascular diseases, diabetes mellitus, and metabolic syndrome, thereby causing persistent damage to multi-system functions (Balakumar et al., 2009).

Drug-induced hyperuricemia (DIH) refers to elevated serum uric acid (SUA) attributable to medication use. Currently, no universally accepted diagnostic criteria or formal definition for DIH has been established. In this review, DIH is operationally defined by adopting the standard biochemical thresholds for hyperuricemia. Specifically, fasting SUA must exceed 420 μmol/L in males and postmenopausal females, or 360 μmol/L in premenopausal females, measured on two separate occasions under standardized dietary conditions (FitzGerald et al., 2020; Multidisciplinary Expert Task Force on Hyperuricemia and Related Diseases, 2017). In addition, a clear temporal association between SUA elevation and the initiation or dose escalation of a suspected drug is required, after exclusion of primary hyperuricemia, renal disease, metabolic syndrome, and other secondary causes. Among the various etiological factors of hyperuricemia, DIH has gradually become a focus of clinical attention due to its strong concealment, complex pathogenic mechanisms, and wide involvement of drug classes. Currently, with the continuous increase in drug types and the popularization of combined medication, the incidence of DIH is rising annually, seriously affecting patients’ medication safety and therapeutic outcomes. Globally, there are significant regional and population differences in the incidence of DIH. In North America, the prevalence of DIH in the general population is approximately 3.9%, while in the UK, drug-induced gout accounts for more than 25% of all gout cases (Ben Salem et al., 2016). High-income countries have a significantly higher incidence of DIH than low- and middle-income countries due to the greater variety of clinical drugs and more widespread use of combined medication. This trend is particularly prominent in elderly patients and populations with chronic diseases receiving combined medication (Piani et al., 2024). Statistical analysis of data from the US Food and Drug Administration Adverse Event Reporting System (FAERS) between 2004 and 2023 revealed a total of 18,531 reported adverse events related to DIH and drug-induced gout worldwide, involving 131 hyperuricemia-inducing drugs and 177 gout-inducing drugs (Zheng et al., 2025).

We conducted a comprehensive literature search across PubMed, Embase, and Web of Science from database inception to 1 January 2026, using the following core keywords and MeSH terms: “hyperuricemia”, “drug-induced hyperuricemia”, “iatrogenic”, “urate transporters”, “purine metabolism”, and “clinical management”. The initial search retrieved 829 records. We excluded duplicates and irrelevant studies through title and abstract screening, leaving 170 articles for full-text review. Subsequently, 94 articles were excluded following full-text assessment. Ultimately, 76 studies were included for qualitative synthesis, with no restrictions on study design. These included clinical trials, observational studies, case series, clinically translatable mechanistic studies, systematic reviews, meta-analyses, and clinical guidelines or consensus statements. This review aims to comprehensively analyze the pathogenic mechanisms of DIH, systematically summarize the characteristics of major causative drugs, and explore effective intervention strategies, providing a scientific basis for optimizing rational clinical medication and promoting individualized treatment.

## 2 Causative drugs acting on uric acid reabsorption and secretion pathways

### 2.1 Diuretics

Diuretics are one of the primary causative factors of DIH. Among them, loop diuretics and thiazide diuretics are significantly associated with an increased risk of hyperuricemia, with loop diuretics exhibiting a stronger association than thiazide diuretics (Leung et al., 2022). These drugs act on the proximal renal tubules, regulate the function of various key transporters, and induce volume contraction, dually promoting uric acid reabsorption and ultimately leading to elevated SUA (Kim and Jun 2022). This effect is dose-dependent, occurring within days of treatment initiation, persisting during long-term administration, and reversing to baseline levels within months after drug discontinuation (Zalem, 2024).

Loop diuretics and thiazide diuretics primarily act on organic anion transporters 1 and 3 (OAT1 and OAT3) located on the basolateral membrane of proximal renal tubule cells. These drugs enter tubular cells via OAT1 and OAT3 and act as competitive substrates for uric acid, inhibiting its secretion and indirectly enhancing uric acid reabsorption (Otani et al., 2017). Additionally, hydrochlorothiazide specifically activates the apical membrane transporter OAT4, significantly increasing uric acid uptake (Raja et al., 2019). Meanwhile, both classes of diuretics inhibit the human voltage-driven drug efflux transporter (NPT4) and multidrug resistance protein 4 (MRP4). NPT4 mediates uric acid secretion, while MRP4 facilitates the final secretion of uric acid from epithelial cells into the tubular lumen. Inhibition of these transporters further blocks uric acid excretion and synergistically promotes reabsorption (Jutabha et al., 2011; Chapa et al., 2020). Notably, genetic variations in the *SLC22A11* gene encoding OAT4 may enhance OAT4 transport capacity, synergizing with diuretic use to increase the risk of hyperuricemia, although this conclusion remains controversial. A large prospective study demonstrated that individuals with a genetic predisposition to hyperuricemia have a similar risk of developing gout after diuretic use compared to those without such a predisposition (Bao et al., 2015).

**FIGURE 1:**
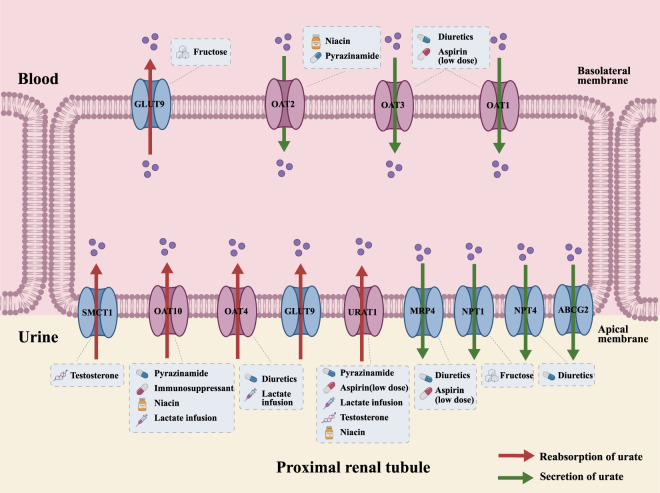
Drug effect on uric acid absorption and secretion pathway. Abbreviations: GLUT9, glucose transporter 9; OAT, organic anion transporter; SMCT1, sodium-dependent monocarboxylate transporter 1; URAT1, urate anion transporter 1; MRP4, multidrug resistance protein 4; NPT, the human voltage-driven drug efflux transporter; ABCG2, ATP-binding cassette superfamily G member 2.

Furthermore, diuretic-induced salt and water loss causes volume contraction, which stimulates uric acid reabsorption in the proximal renal tubules. Specifically, thiazide diuretic-induced volume contraction augments proton (H^+^) secretion through the apical membrane Na^+^/H^+^ exchanger 3 (NHE3) in renal proximal tubular epithelial cells. The resultant elevation of intracellular pH further promotes uric acid uptake via OAT4, which relies on a urate-hydroxyl anion exchange pathway (Kanai, 2008). Furosemide can also induce hyperlactatemia, indirectly inhibiting renal tubular uric acid excretion (Zheng et al., 2025) (Figure 1).

Indapamide, a thiazide-like diuretic widely used in hypertension management, competitively inhibits OAT1/OAT3-mediated uric acid secretion and indirectly enhances reabsorption secondary to volume contraction, as do traditional thiazides. Pooled data from three randomized double-blind trials (n = 1,195) revealed that sustained-release indapamide at 1.5 mg/day produced only a slight transient increase in SUA during short-term therapy. Over 12 months of follow-up, SUA levels returned to baseline, indicating a favorable metabolic profile for sustained-release formulations (Weidmann, 2001). Meta-analyses showed comparable effects of indapamide and hydrochlorothiazide on SUA, despite indapamide’s greater antihypertensive efficacy (Roush et al., 2015). In hypertensive patients with pre-existing hyperuricemia, combination therapy with losartan effectively attenuated the hyperuricemic effect of indapamide by increasing fractional urate excretion (Sun and Wang, 2024). For hypertensive patients with concomitant hyperuricemia or gout, low-dose sustained-release indapamide may be preferable, with combination therapy using uricosuric agents or alternative antihypertensive agents such as losartan recommended to mitigate metabolic risk.

In contrast to loop and thiazide diuretics, the effects of potassium-sparing agents on uric acid metabolism are heterogeneous and population-specific. In patients with heart failure and preserved ejection fraction, a substudy of the TOPCAT trial found no significant change in SUA after 12 months of spironolactone therapy compared with placebo (Myhre et al., 2020). This finding suggests that spironolactone exerts a neutral effect on uric acid in the general heart failure population. However, in patients with chronic kidney disease, low-dose spironolactone has been shown to increase SUA levels. In this population, spironolactone loses its mild inhibitory effect on urate transporter 1 (URAT1) and instead promotes uric acid reabsorption by reducing renal blood flow and increasing lactate production (Cabrera et al., 2014). Additionally, combined use of spironolactone and loop diuretics in heart failure patients leads to a significant increase in SUA. This synergistic effect is closely related to loop diuretic-induced changes in the tubular microenvironment, which convert spironolactone from a URAT1 inhibitor to an activator (Felker et al., 2020) (Table 1). Given these conflicting data, a risk-stratified clinical approach is recommended. For patients with heart failure or resistant hypertension who have preserved renal function, spironolactone remains a cornerstone therapy according to current guidelines (Jones et al., 2025; Maddox et al., 2024). Clinicians should obtain a baseline SUA level and monitor it periodically during follow-up. For patients with CKD or those receiving concomitant loop diuretics, the risk of hyperuricemia is substantially higher. In these cases, more frequent monitoring is warranted. If SUA rises significantly or gout develops, switching to finerenone should be considered (Wang et al., 2025).

**TABLE 1:** Common drugs targeting uric acid reabsorption and secretion pathways.

Drug category	Causative drugs	Suggested mechanism	References
Diuretic	Furosemide	Increased uric acid reabsorption Decreased uric acid secretion Volume contraction	Kim and Jun (2022)
	Hydrochlorothiazide	Increased uric acid reabsorption Decreased uric acid secretion	Raja et al. (2019)
	Indapamide	Increased uric acid reabsorption	Roush et al. (2015)
	Spironolactone	Controversial	Cabrera et al. (2014)
Anti-tubercular drugs	Pyrazinamide	Increased uric acid reabsorption Decreased uric acid secretion	Pham et al. (2014)
	Ethambutol	Reduced urate excretion	Louthrenoo et al. (2015)
Non-steroidal anti-inflammatory drugs	Aspirin (low dose)	Increased uric acid reabsorption Decreased uric acid secretion	Wang et al. (2014)
Immunosuppressant	Cyclosporine	Increased uric acid reabsorption Reduced urate excretion	Laine and Holmberg (1996)
	Tacrolimus	Reduced urate excretion	André et al. (2022)
Sodium Lactate Solution	Lactate infusion	Increased uric acid reabsorption	Kane et al. (2025)
Sex hormones	Testosterone	Increased uric acid reabsorption	Hosoyamada et al. (2010)
Lipid-lowering drugs	Niacin	Increased uric acid reabsorption Decreased uric acid secretion	Zhu et al. (2025)

### 2.2 Antituberculosis drugs

Antituberculosis drugs are another important class of agents causing DIH. Among them, pyrazinamide is the primary drug that induces hyperuricemia in tuberculosis patients, with highly variable incidence ranging from 43% to 84.5% (Putra et al., 2024; Taki et al., 2008). Although 84.5% of these patients had hyperuricemia, only 4.42% experienced joint pain, and no patient discontinued pyrazinamide treatment due to hyperuricemia. This finding provides an important reference for clinical decision-making (Louthrenoo et al., 2015). Additionally, gender differences are evident in pyrazinamide-induced hyperuricemia, with a higher incidence in male patients than in females (Alberio et al., 2025). A study by Khanna (1980) indicated that ethambutol can also significantly increase SUA, with elevated levels observed 24 h after a single dose, peaking at 2–4 weeks of treatment. SUA returns to normal after drug discontinuation and recurs upon re-administration, suggesting a direct drug-dependent interference with uric acid excretion.

Both pyrazinamide and ethambutol interfere with uric acid metabolism primarily in the proximal renal tubules. Pyrazinamide promotes uric acid reabsorption and inhibits secretion through well-defined transporter regulatory mechanisms (Pham et al., 2014), ethambutol primarily reduces uric acid excretion, although its specific molecular mechanisms remain not yet fully elucidated (Louthrenoo et al., 2015). The promotion of uric acid reabsorption by pyrazinamide depends on its active metabolite pyrazinoate (PZA). In the pathway of increased uric acid reabsorption, PZA activates the URAT1 transporter on the apical membrane of proximal renal tubules through a trans-stimulatory effect, significantly enhancing urate reabsorption from the tubular lumen into epithelial cells (Enomoto et al., 2002; Sato et al., 2011). This mechanism is supported by genetic evidence, as individuals with loss-of-function mutations in the URAT1 gene exhibit a blunted hyperuricemic response to pyrazinamide (Mandal and Mount, 2015). Additionally, PZA can exchange with the organic anion/dicarboxylate exchanger OAT10 in the proximal renal tubules, further increasing OAT10-mediated urate uptake and synergistically enhancing the reabsorptive effect (Toyoda et al., 2022) (Table 1).

Pyrazinamide also exacerbates hyperuricemia by inhibiting the uric acid secretion pathway. It specifically inhibits the OAT2 transporter expressed on the basolateral membrane of proximal renal tubules, directly blocking uric acid secretion. When combined with increased reabsorption, this dual effect significantly elevates SUA levels (Sato et al., 2010).

### 2.3 Low-dose aspirin

Aspirin exerts a dose-dependent effect on uric acid metabolism. Low-dose aspirin (75–150 mg/d) induces hyperuricemia by inhibiting uric acid excretion, whereas high-dose aspirin (≥3,000 mg/d) promotes uric acid excretion (Ben Salem et al., 2024).

In the pathway of increased uric acid reabsorption, salicylate acts as an exchange substrate for the urate-monocarboxylate exchanger URAT1 at low doses, significantly promoting urate reabsorption from the tubular lumen into epithelial cells via URAT1-mediated exchange (Nakanishi et al., 2013). Even at extremely low doses, salicylate inhibits the activity of MRP4 on the apical membrane of proximal renal tubules, indirectly enhancing uric acid reabsorption (Halperin and Woodward, 2021). Furthermore, salicylate exacerbates hyperuricemia through interactions with the basolateral organic anion transporters OAT1 and OAT3 in the proximal renal tubules (Wang et al., 2014). Inhibition of OAT1 and OAT3 directly impairs the renal uric acid secretion process, which, combined with the promotion of uric acid reabsorption, further elevates SUA levels (Table 1).

A recent study confirmed that low-dose aspirin is an independent risk factor for hyperuricemia, with a lower dose associated with a higher risk, thereby fully validating its dose-dependent hyperuricemic effect (Zhu et al., 2024). Notably, the impact of low-dose aspirin on uric acid is age- and renal function-dependent. In individuals aged 40–50 years and those with renal insufficiency, a lower dose (75 mg) is more likely to cause elevated uric acid (Zhu B. et al., 2023).

### 2.4 Immunosuppressants

Both cyclosporine and tacrolimus are calcineurin inhibitor immunosuppressants. Experimental studies have confirmed that cyclosporine significantly increases urate uptake by enhancing the function of the OAT10 transporter in the proximal renal tubules, directly promoting uric acid reabsorption. Additionally, cyclosporine indirectly exacerbates uric acid excretion disorders by constricting renal afferent arteriole, leading to decreased glomerular filtration rate and reduced uric acid filtration and excretion (El-Sheikh et al., 2013). When combined with diuretics, cyclosporine synergizes with diuretic-induced volume depletion to further stimulate uric acid reabsorption in the proximal renal tubules, enhancing the hyperuricemic effect (Laine and Holmberg, 1996).

Tacrolimus also affects uric acid metabolism primarily by reducing uric acid excretion in the proximal renal tubules, ultimately inducing hyperuricemia, although its specific core targets and molecular mechanisms remain incompletely understood (André et al., 2022) (Table 1). Compared with cyclosporine, tacrolimus exerts a relatively milder hyperuricemic effect, resulting in a lower incidence of related complications in clinical practice. Studies have reported that up to 80% of kidney transplant recipients develop hyperuricemia, with cyclosporine exerting a more prominent adverse effect on uric acid metabolism than tacrolimus (Gomez-Coral, 2025). Therefore, close monitoring of SUA levels is necessary in kidney transplant recipients receiving cyclosporine. In addition to controlling immune rejection, clinicians should actively intervene to manage hyperuricemia. Medication regimens should be adjusted based on patients’ renal function to reduce the risk of hyperuricemia (Junk et al., 2025).

### 2.5 Sodium lactate solution

Sodium lactate solution offers significant advantages in the resuscitation of critically ill patients. However, high-dose infusion may induce hyperuricemia by reducing urinary uric acid excretion and impairing uric acid clearance (Ped et al., 2025). A study by Drabkin et al. (2019) also indicated that lactate infusion causes fluctuations in sodium metabolism in the human body, thereby interfering with uric acid excretion through multiple pathways such as renal blood flow and transport systems. Long-term or high-dose infusion increases the risk of hyperuricemia.

The hyperuricemic effect of sodium lactate solution primarily relies on the specific interaction between lactate and key transporters in the proximal renal tubules. As an exchange substrate for URAT1, lactate directly stimulates URAT1-mediated uric acid reabsorption after infusion. Additionally, lactate indirectly enhances uric acid reabsorption and inhibits uric acid secretion by interfering with the organic anion transporters OAT4 and OAT10 in the proximal renal tubules (Kane et al., 2025) (Table 1).

### 2.6 Testosterone

Early cross-sectional studies suggested a negative correlation between testosterone and hyperuricemia. A study by Bac et al. (2023) indicated that low testosterone status exacerbates the development of hyperuricemia, which may be closely associated with increased insulin resistance. Insulin resistance activates the renal uric acid reabsorption transporter URAT1, leading to increased uric acid reabsorption. Meanwhile, low testosterone is often accompanied by metabolic syndrome manifestations such as central obesity, which collectively promote hyperuricemia. This association is particularly significant in males with type 2 diabetes (Bac et al., 2023). A large-scale cross-sectional study based on NHANES data also confirmed an independent negative correlation between serum total testosterone and SUA levels (Han et al., 2021). However, other studies have provided conflicting evidence. These studies suggest that higher serum total testosterone levels are independently associated with higher SUA levels and a higher risk of hyperuricemia (Tsai et al., 2022). In animal experiments, orchiectomized male rats exhibited decreased SUA levels, which reversed after testosterone supplementation (Ling et al., 2020). Testosterone can specifically induce the expression of sodium-dependent monocarboxylate transporter 1 (Smct1) in the proximal renal tubules, synergizing with URAT1 to promote urate reabsorption in the proximal renal tubules. This is a key potential molecular mechanism underlying testosterone-induced hyperuricemia in males (Hosoyamada et al., 2010). URAT1 mediates the exchange and uptake of urate and anions on the luminal side, and the induced expression of Smct1 enhances the efficiency of this process, ultimately reducing renal uric acid excretion and increasing SUA levels (Lu et al., 2013) (Table 1). Additionally, testosterone increases SUA levels by promoting protein synthesis and nucleic acid turnover rate, and enhancing the activity of hypoxanthine-guanine phosphoribosyltransferase. A multicenter study further confirmed that the prevalence of hyperuricemia in males is significantly higher than that in females of the same age (Pang et al., 2021). Notably, testosterone may exert different effects under different physiological conditions. This finding is consistent with the results of a prospective study, which indicated that free and bioavailable testosterone may be positively correlated with the risk of elevated uric acid, while total testosterone may have a beneficial effect on uric acid metabolism through anti-inflammatory and metabolic improvement mechanisms (Jiang et al., 2024).

### 2.7 Niacin

Niacin (nicotinic acid) is a commonly used lipid-lowering agent and one of the high-risk drugs for inducing hyperuricemia. A real-world data analysis reported that the incidence of hyperuricemia in niacin users was 2.34%, significantly higher than that in the control group using other lipid-lowering drugs (Zheng et al., 2025).

Niacin salts promote uric acid reabsorption through specific interactions with URAT1 (Fujita et al., 2023). In addition, niacin salts are exchanged via OAT10, significantly increasing OAT10-mediated urate uptake (Ohtsu et al., 2022). Niacin also specifically inhibits the OAT2 transporter on the basolateral membrane of proximal renal tubules, thereby inhibiting uric acid secretion and elevating SUA levels (Mathialagan et al., 2020) (Table 1). Additionally, niacin exacerbates hyperuricemia by increasing uric acid synthesis. Niacin itself and its amide derivative nicotinamide can activate phosphoribosyl pyrophosphate synthetase (PRPP synthetase), the rate-limiting enzyme in the *de novo* purine synthesis pathway, increasing intracellular PRPP concentration, significantly accelerating purine nucleotide synthesis, and increasing uric acid precursors. This is an important supplementary mechanism underlying niacin-induced hyperuricemia (Gouni-Berthold and Berthold, 2013) (Table 2). A recent study suggested that the level of nicotinuric acid, the main metabolite of niacin, in the urine of gout patients is 6.5 times higher than that in patients with asymptomatic hyperuricemia (Zhu et al., 2025). Therefore, niacin is both an exogenous drug that increases uric acid levels and a key endogenous metabolic hub leading to gout. Urinary levels of niacin and nicotinuric acid not only serve as non-invasive biomarkers for predicting gout risk but also indicate that their associated metabolic pathways may represent potential therapeutic targets for preventing future gout attacks.

**TABLE 2:** Common drugs that lead to an increase in uric acid synthesis.

Drug category	Causative drugs	Suggested mechanism	References
Cytotoxic chemotherapeutic	Bleomycin Rituximab Ibrutinib Bortezomib	Massive destruction of tumor cells	Sanagawa et al. (2020)
Biological agents	Filgrastim	Massive hematopoietic cell apoptosis	D'Souza et al. (2008)
Non-glucose carbohydrates	Fructose	Enhancing nucleotide turnover and synthesis	Zhang et al. (2022)
	Glycerol	Stimulate uric acid production	Petrie et al. (2013)
	Xylitol	Impair red blood cell glycolysis	Meyer-Gerspach et al. (2021)
	Sorbitol	Affecting the level of adenosine phosphate in the liver	Delannoy et al. (2025)
Lipid-lowering drugs	Nicotinic acid	Activate phosphoribosyl pyrophosphate synthetase	Gouni-Berthold and Berthold (2013)

## 3 Causative drugs primarily acting on uric acid production pathways

### 3.1 Cytotoxic chemotherapeutic drugs and biological agents

Cytotoxic drugs, including bleomycin, rituximab, and ibrutinib, are key causative agents acting on the uric acid production pathway and major contributors to DIH. These drugs potently destroy tumor cells, leading to the massive release of intracellular nucleic acids and purine nucleotides into the bloodstream, thereby triggering tumor lysis syndrome (TLS) and a significant surge in uric acid production (Lupuşoru et al., 2022). Notably, in addition to traditional cytotoxic chemotherapeutic drugs, novel chemotherapeutic agents such as dexamethasone, zoledronic acid, thalidomide, or bortezomib may also induce TLS-related hyperuricemia through the same core mechanism (Sanagawa et al., 2020) (Table 2).

**FIGURE 2:**
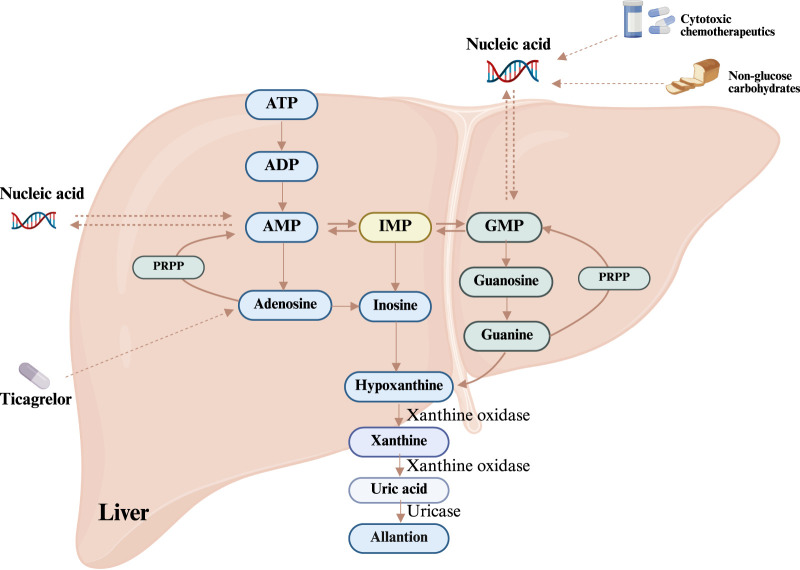
Effect of drugs on the uric acid synthesis pathway. Abbreviations: ATP, adenosine triphosphate; ADP, adenosine diphosphate; AMP, adenosine monophosphate; IMP, inosine monophosphate; GMP, guanosine monophosphate; PRPP, 5-phosphoribosyl-1-pyrophosphate.

In clinical practice, cytotoxic drugs are often combined with the biological agent filgrastim to promote granulocyte recovery after chemotherapy-induced myelosuppression. Studies have shown that this combination further increases the risk of hyperuricemia (Sarno, 2013). Filgrastim stimulates the proliferation and differentiation of bone marrow hematopoietic stem cells into the granulocyte lineage. During this process, nucleic acid synthesis and catabolism in hematopoietic cells are concomitantly enhanced, and apoptosis of numerous hematopoietic cells releases purine nucleotides, which are ultimately converted into uric acid via xanthine oxidase catalysis. This mechanism synergizes with the pathway of purine release via tumor cell apoptosis induced by cytotoxic drugs, significantly increasing total purine metabolism and exacerbating hyperuricemia (D'Souza et al., 2008) (Figure 2).

### 3.2 Non-glucose carbohydrates

Non-glucose carbohydrates, including fructose, glycerol, xylitol, and sorbitol, are used as glucose substitutes in total parenteral nutrition in clinical practice. Their metabolism can induce hyperuricemia through multisite, multipathway mechanisms. The core sites of action of these substances on uric acid metabolism include the liver, kidneys, and systemic cells (e.g., red blood cells, parenchymal cells). Core targets involve the purine metabolism pathway, glycolytic pathway, and uric acid transport-related pathways. Key transporters include glucose transporter 9 (GLUT9) and human inorganic phosphate transporter 1 (NPT1) (Kawakami et al., 2023). Their pathogenic mechanisms mainly involve enhanced purine metabolism or accelerated degradation, as well as inhibition of uric acid excretion (Table 2).

Fructose increases uric acid production by enhancing nucleotide turnover and synthesis, inhibits uric acid excretion by increasing blood lactate and inducing insulin resistance or hyperinsulinemia, and promotes renal tubular urate reabsorption via GLUT9. Accordingly, genetic variations in the *SLC2A9* gene encoding GLUT9 and the *SLC17A1* gene encoding NPT1 can alter individual sensitivity to fructose (Zhang et al., 2022; Batt et al., 2014). Glycerol induces hyperuricemia by stimulating uric acid production, reducing cellular ATP levels, and increasing glycerol-3-phosphate, uric acid production is inversely correlated with ATP levels (Petrie et al., 2013). Xylitol primarily promotes purine degradation by impairing red blood cell glycolysis, thereby increasing SUA, hypoxanthine, and xanthine concentrations (Meyer-Gerspach et al., 2021). Sorbitol is metabolized to fructose in the liver, indirectly increasing uric acid production by affecting hepatic adenosine phosphate levels (Delannoy et al., 2025).

## 4 Other potential causative drugs

Beyond the extensively characterized drug classes discussed above, numerous other pharmacological agents have been implicated in DIH (Table 3).

**TABLE 3:** Other drugs and their suggested mechanisms that cause hyperuricemia.

Drug category	Causative drugs	Suggested mechanism	References
Beta-blockers	Metoprolol	Reduce renal blood flow and glomerular filtration rate Decrease uric acid excretion	Leung et al. (2022)
Peptide hormones	Insulin	Reduce uric acid excretion Increased uric acid reabsorption	MacFarlane et al. (2015)
Immunosuppressant	Azathioprine	Increased uric acid production	Ben Salem et al. (2016)
Immunomodulators	Lenalidomide (high-dose)	Increased uric acid production	Andritsos et al. (2008)
Antiplatelet aggregation agent	Ticagrelor	Increased uric acid production	Butler and Teng (2011)
Proton pump inhibitors	Omeprazole	Decrease uric acid excretion	Zhu et al. (2023b)
Parathyroid hormone analogues	Teriparatide	Unknown	Hoong et al. (2025)
Antiviral drug	Didanosine	Increased uric acid production	Walker et al. (2006)
	Tenofovir	Unknown	Datta et al. (2022)
Antibiotics	Fluoroquinolones	Decrease uric acid excretion	Mandovra et al. (2020)
Dopamine precursor drugs	Levodopa	Unknown	Songsomboon et al. (2020)

Several drug classes appear to elevate SUA primarily through impaired renal elimination. Beta-adrenergic receptor antagonists, typified by metoprolol, may reduce glomerular filtration rate through decreased cardiac output and renal perfusion pressure, thereby diminishing the filtered urate load available for excretion (Leung et al., 2022). However, it should be noted that many commercially available beta-blocker formulations are combined with thiazide diuretics, which independently and potently induce hyperuricemia through direct tubular effects, and evidence attributing hyperuricemia specifically to beta-blocker monotherapy remains limited. Insulin activates multiple renal urate transporters, particularly GLUT9, the dominant basolateral exit pathway for filtered urate in the proximal tubule, thereby enhancing urate reabsorption. Insulin also stimulates proximal tubular sodium reabsorption, which may indirectly promote urate retention (MacFarlane et al., 2015).

Certain agents elevate SUA by expanding the purine substrate pool or accelerating nucleotide degradation. Azathioprine, a purine analog prodrug of 6-mercaptopurine, interferes directly with *de novo* and salvage purine pathways, increasing nucleotide turnover and downstream uric acid generation (Ben Salem et al., 2016). Notably, co-prescription with xanthine oxidase inhibitors requires caution, as inhibition of 6-mercaptopurine catabolism shunts metabolism toward cytotoxic thioguanine nucleotides, resulting in severe myelosuppression (Madrazo et al., 2021). Should co-administration prove unavoidable, the azathioprine dose must be reduced to 25%–50% of standard, with intensive hematological monitoring (Logan et al., 2020). High-dose lenalidomide can induce hyperuricemia through increased cell turnover and tumor lysis syndrome–like pathophysiology, particularly in patients with hematologic malignancies (Andritsos et al., 2008). Ticagrelor elevates circulating adenosine by inhibiting erythrocyte adenosine reuptake. Subsequent catabolism via xanthine oxidase generates increased hypoxanthine and xanthine, which are metabolized to uric acid via xanthine oxidase, thereby increasing SUA (Butler and Teng, 2011). Didanosine, a synthetic purine nucleoside analog used in HIV therapy, has been associated with asymptomatic hyperuricemia in clinical trials and post-marketing surveillance (Walker et al., 2006). Its structural similarity to endogenous purines suggests direct interference with purine metabolic pathways, although the precise enzymatic targets remain undefined.

For several drug classes, the association with hyperuricemia is recognized clinically, but the underlying molecular mechanisms remain poorly elucidated. Population-based case-control studies have linked proton pump inhibitors such as omeprazole to incident gout. The biological basis for this association remains elusive. Hypothesized mechanisms include alterations in gut microbiota composition and intestinal uric acid handling, yet direct experimental evidence is lacking (Zhu KJ. et al., 2023). Teriparatide increased SUA concentrations in approximately 3% of treated patients versus 1% of placebo recipients in randomized osteoporosis trials. The specific renal or metabolic mechanisms underlying this observation remain undefined (Hoong et al., 2025). Tenofovir disoproxil fumarate is associated with proximal renal tubular dysfunction and Fanconi syndrome, which may secondarily alter uric acid handling, yet direct evidence linking this agent to hyperuricemia through a specific transporter-mediated mechanism remains limited (Datta et al., 2022). Fluoroquinolones have been associated with new-onset joint pain in some patient populations, and isolated reports suggest a possible link to hyperuricemia, but their precise effects on renal urate transporters remain undefined (Mandovra et al., 2020). Levodopa has been reported to interfere with colorimetric uric acid assays, and any clinical effect on endogenous uric acid metabolism remains undefined (Songsomboon et al., 2020).

## 5 Intervention strategies for drug-induced hyperuricemia

### 5.1 Drug adjustment regimens

**FIGURE 3:**
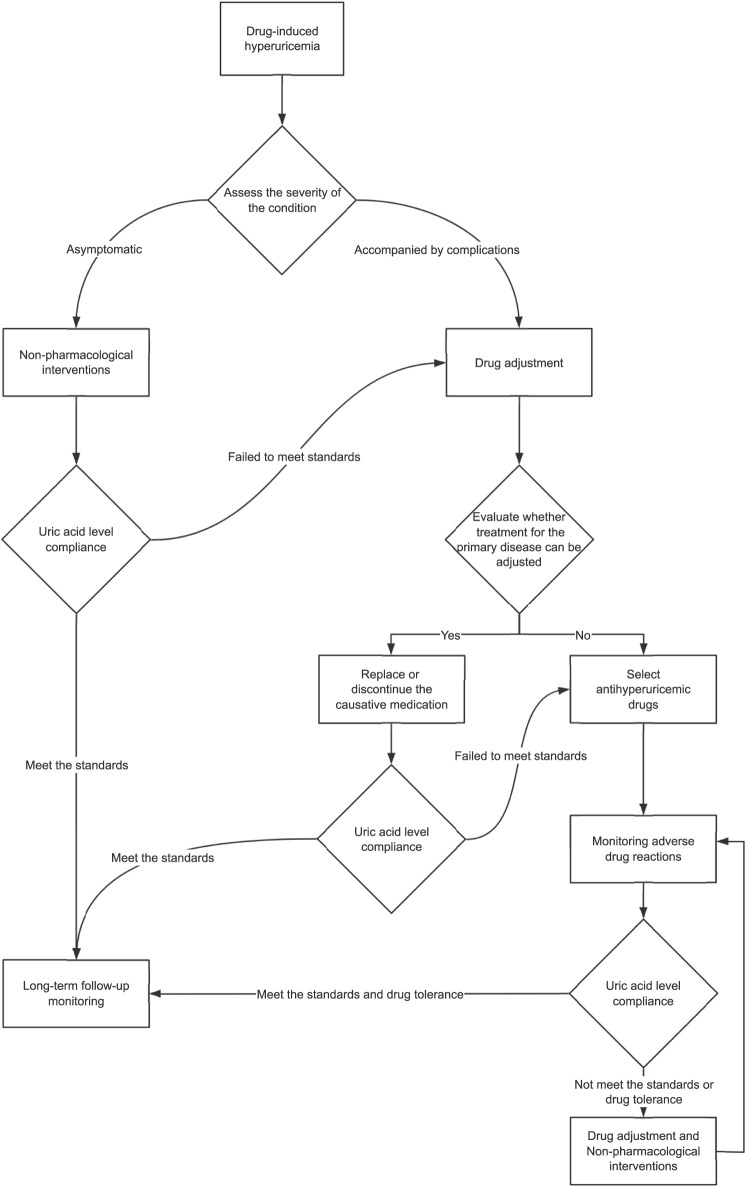
Intervention strategies for drug-induced hyperuricemia.

Drug adjustment is the fundamental strategy for managing DIH. The primary principle is to discontinue the causative drug whenever possible or select alternative drugs with similar pharmacological effects but minimal impact on uric acid metabolism, while ensuring therapeutic efficacy for the underlying condition. For example, in patients with hypertension complicated by hyperuricemia, angiotensin II receptor blockers with uricosuric effects such as losartan are recommended, and thiazide diuretics should be avoided (Koziolova and Chernyavina, 2022; Wu et al., 2025). In kidney transplant recipients who develop hyperuricemia, cyclosporine can be switched to tacrolimus. Clinical studies have shown that this switch can effectively ameliorate refractory gout (André et al., 2022). In tuberculosis patients, although pyrazinamide often causes hyperuricemia, most patients do not require discontinuation of the drug. Clinical practice recommends close monitoring as the primary approach, and adjustment of the antituberculosis regimen should be considered only in cases of severe gout symptoms, tophi, or renal impairment (Putra et al., 2024). Notably, drug adjustment requires balancing the therapeutic needs of the primary disease with the risk of hyperuricemia, necessitating individualized decision-making (Figure 3).

### 5.2 Application of urate-lowering agents

When drug adjustment fails to effectively control hyperuricemia or treatment of the primary disease cannot be interrupted, the addition of urate-lowering agents should be considered (Table 4). Xanthine oxidase inhibitors (e.g., allopurinol and febuxostat) exert their effects by inhibiting uric acid production, and are particularly suitable for the prevention and treatment of TLS (Gliozzi et al., 2016). It is important to note that these drugs have potential safety concerns during use. Allopurinol may cause hypersensitivity reactions, which have been shown to be associated with the HLA-B*5801 allele (Tassaneeyakul et al., 2009). Febuxostat may increase the risk of death in patients with cardiovascular diseases (White et al., 2018). The novel drug topiroxostat is a highly selective non-purine xanthine oxidoreductase (XOR) inhibitor. Due to its minimal impact on oxidative stress, it not only lowers uric acid but also has potential renoprotective effects (Horino et al., 2017). Uricosuric agents (e.g., benzbromarone, probenecid) increase uric acid excretion by inhibiting renal tubular uric acid reabsorption, but clinicians should be aware of their potential to increase the risk of urinary tract stones (Bach and Simkin, 2014). Among them, although benzbromarone has a significant urate-lowering effect, it carries a risk of severe liver injury, requiring close monitoring of liver function during clinical use (Xu et al., 2021). Additionally, the novel drug dotinurad, a highly selective URAT1 inhibitor (Osonoi et al., 2025), can specifically inhibit renal uric acid reabsorption without affecting intestinal ABCG2-mediated uric acid excretion. It remains effective in patients with combined hepatic and renal impairment or hypertension without dose adjustment, and has good long-term safety, making it a preferred uricosuric agent in clinical practice. However, approximately 3% of gout patients fail to achieve the target SUA level with oral urate-lowering agents. For these refractory cases, international guidelines recommend uricase preparations as second-line agents. Rasburicase, a recombinant uricase, can directly convert uric acid into soluble allantoin, offering unique advantages in the emergency management of TLS (Cammalleri and Malaguarnera, 2007). In refractory chronic gouty arthritis, low-dose rasburicase effectively reduces SUA levels, decreases tophaceous burden, and is generally well tolerated (Zeng et al., 2022). However, rasburicase is absolutely contraindicated in patients with glucose-6-phosphate dehydrogenase (G6PD) deficiency. The enzymatic conversion of uric acid to allantoin generates hydrogen peroxide, which can trigger severe oxidative hemolysis and methemoglobinemia in G6PD-deficient individuals. Regulatory agencies mandate screening for G6PD deficiency prior to administration, particularly in high-risk ethnic groups (Hammami et al., 2024). A systematic review reported that affected patients typically develop hemolysis or methemoglobinemia within 24 h, with a median methemoglobin level of 10%; although most recover with supportive care, fatal cases have occurred (Hammami et al., 2024). If these complications arise, rasburicase must be permanently discontinued. In G6PD-deficient patients requiring urate-lowering therapy, allopurinol or febuxostat should be used instead (Stone et al., 2024).

**TABLE 4:** Common drugs for reducing uric acid levels.

Drug category	Therapeutic drugs	Main mechanism	Safety profile	References
Xanthine oxidase inhibitor	Allopurinol	Inhibiting XO	Cause allergic reactions	Tassaneeyakul et al. (2009)
	Febuxostat	Inhibiting XO	Increase the risk of death	White et al. (2018)
	Topiroxostat	Highly selective inhibition of XO	Potential renal protective effect	Horino et al. (2017)
Uricosuric agents	Probenecid	Inhibit the reabsorption of uric acid Increase uric acid excretion	Increase the risk of urinary stones	Bach and Simkin (2014)
	Benzbromarone		Cause severe liver damage	Xu et al. (2021)
	Dotinurad	Inhibit uric acid transporter 1 Reduce uric acid reabsorption	Favorable long-term safety profile	Osonoi et al. (2025)
Uricase preparations	Rasburicase	Catalyzes urate into soluble allantoin	Good tolerability	Cammalleri and Malaguarnera (2007)
TCM compound preparations	Huazhuo Sanjie Chubi Decoction	Multi-target effect	Mild toxic and side effects	Zhong et al. (2026)
	SiMiao Wan	Multi-target effect	Mild toxic and side effects	Guo et al. (2025)
	Wuling Capsule	Modulates urate transporters	Mild toxic and side effects	Li et al. (2025)
TCM herb extracts	Quercetin	Multi-target effect	Mild toxic and side effects	Zhang et al. (2026)
	Piper kadsura extract	Inhibition of GLUT9 expression Reduce oxidative stress	Mild toxic and side effects	Pan et al. (2025)
	Astragali Radix extract	Regulate PI3K/AKT signaling pathway	Mild toxic and side effects	Zhang et al. (2023)

Some traditional Chinese medicine (TCM) preparations have shown potential in the treatment of mild to moderate DIH. Preclinical and small-scale clinical studies have reported that various TCM interventions, including single herbs and compound formulas, may ameliorate hyperuricemia through multi-targeted effects, symptomatic relief, and favorable tolerability profiles, thereby offering a potential alternative or adjunctive treatment option (Gong, 2024). A growing body of evidence suggests that ABCG2 may represent a key target for TCMs exerting comprehensive intestinal-renal therapeutic effects and systemic anti-gout actions, although the precise regulatory mechanisms remain incompletely elucidated. Notably, although no chemical drugs specifically targeting ABCG2 are currently marketed, numerous TCM-derived compounds have been reported to achieve synergistic urate-lowering and anti-inflammatory effects by modulating multiple targets, including ABCG2, XOR, and URAT1 (Table 4). For example, Huazhuo Sanjie Chubi Decoction not only alleviates local inflammation in gout patients but also lowers uric acid through ingredients such as Clematidis Radix, Cortex Phellodendri, and Smilax glabra Roxb, enabling comprehensive prevention and treatment of gout (Zhong et al., 2026). Among these ingredients, Cortex Phellodendri reduces uric acid production by inhibiting xanthine oxidase activity (Xu et al., 2022), whereas Smilax glabra Roxb regulates intestinal flora to ameliorate purine metabolic disturbances (Shi et al., 2025). Additionally, other compound TCMs such as SiMiao Wan can effectively lower SUA by inhibiting the IL-1β/NF-κB pathway, upregulating intestinal ABCG2 expression, modulating the URAT1/OAT1 balance, and improving insulin resistance (Guo et al., 2025). Wuling Capsule alleviates hyperuricemia and uric acid-induced proximal tubular epithelial cell injury by regulating uric acid transporters (Li et al., 2025). Regarding bioactive constituents and herbal extracts, the urate-lowering mechanisms of several single compounds have been characterized in preclinical models. Quercetin synergistically lowers uric acid and attenuates inflammation by inhibiting XOR activity, downregulating URAT1 and GLUT9 expression, and suppressing NLRP3 inflammasome activation (Zhang et al., 2026). Piper kadsura extract primarily reduces SUA levels and relieves gouty arthritis by inhibiting renal GLUT9 expression and mitigating oxidative stress (Pan et al., 2025). Furthermore, Astragali Radix extract promotes uric acid excretion by regulating the phosphatidylinositol 3-kinase/protein kinase B (PI3K/AKT) signaling pathway to modulate renal urate transporter function (Zhang et al., 2023).

Several important limitations must be acknowledged when considering TCM for DIH. First, robust evidence from large-scale multicenter randomized controlled trials with rigorous blinding and predefined hard endpoints remains scarce. Most existing clinical studies are small single-center investigations with significant methodological heterogeneity and unclear risk of bias. Second, traditional Chinese medicine preparations lack standardization across raw material sourcing, processing methods, extraction protocols, and quality control, leading to considerable batch-to-batch variability in active constituent profiles. Third, the precise active ingredients, optimal dosing regimens, and long-term safety profiles of many TCM interventions remain poorly defined, and pharmacovigilance data regarding herb-drug interactions remain insufficient. Fourth, the mechanisms underlying many TCM effects have been elucidated primarily in animal models or *in vitro* systems, and their translational validity to human DIH requires rigorous confirmation. Therefore, although TCM may serve as a potentially valuable adjunctive therapeutic option, its integration into evidence-based international guidelines will require further high-quality clinical investigation, standardized manufacturing practices, and comprehensive safety profiling.

### 5.3 Non-pharmacological interventions

Non-pharmacological interventions constitute an integral component of the comprehensive management of DIH. Lifestyle modifications primarily include dietary restriction of high-purine food intake, reduced alcohol consumption, and increased fluid intake to enhance uric acid excretion. Dietary adjustments should specifically target fructose intake control, as fructose increases uric acid production and reduces excretion through dual mechanisms (Zhang et al., 2020). Moderate exercise helps control weight and improves insulin resistance, thereby indirectly promoting uric acid metabolism. In recent years, gut microbiota regulation has emerged as a novel target for hyperuricemia treatment. Probiotics and prebiotics have shown potential value by regulating intestinal uric acid transport and purine metabolism (Yang et al., 2025; Cui et al., 2025). Additionally, natural products such as apigenin and mangiferin regulate uric acid metabolism through multi-targeted mechanisms, offering novel therapeutic avenues for hyperuricemia prevention and management (Xu et al., 2025; Shi et al., 2023). Patient education, as a cornerstone of non-pharmacological interventions, improves treatment outcomes by improving patient awareness of DIH and enhancing treatment adherence.

## 6 Conclusion

Drug-induced hyperuricemia, a prominent issue at the intersection of clinical pharmacovigilance and metabolic health, has become a critical consideration in improving treatment effectiveness and safety. Centered on the dual pillars of pharmacological mechanisms and clinical translation, this review systematically elucidates the pathophysiological patterns of DIH via two principal pathways: uric acid transporter dysfunction and purine metabolic imbalance. We integrate multi-dimensional strategies including drug adjustment, targeted intervention, and non-pharmacological management, providing a systematic reference for optimizing pharmacotherapy and clinical decision-making.

From a mechanistic perspective, the identification of targets such as transporters (URAT1, OAT family, GLUT9) and metabolic enzymes (PRPP synthetase, xanthine oxidase) has challenged the notion that DIH is an unavoidable consequence of pharmacotherapy. The elucidation of transporter-mediated pathogenic mechanisms of diuretics, antituberculosis drugs, low-dose aspirin, and other agents provides a molecular basis for therapeutic substitution (e.g., losartan in place of thiazide diuretics). The pathogenic pathways of chemotherapeutic drugs and other agents via disruption of purine metabolism inform decisions regarding prophylactic urate-lowering therapy prior to chemotherapy. Additionally, the intervention strategies summarized in this review highlight the dual emphasis on mechanism-driven innovation and clinical applicability. The development of novel urate-lowering agents directly addresses the safety shortcomings of traditional drugs. TCM preparations achieve synergistic urate-lowering and anti-inflammatory effects by regulating multiple targets such as ABCG2 and URAT1, expanding treatment options for patients with mild-to-moderate DIH. Non-pharmacological strategies such as gut microbiota modulation and natural product-based interventions provide supplementary options for drug-intolerant patients. Collectively, these strategies suggest that DIH management requires balancing treatment of the underlying condition against the risk of iatrogenic hyperuricemia, thereby shifting the clinical paradigm from passive adverse event monitoring to proactive avoidance of high-risk medications.

However, controversies and limitations persist in translating these findings to clinical practice. The therapeutic effects of spironolactone exhibit population heterogeneity between patients with CKD and the general population, and genetic variations such as *SLC22A11* and *SLC2A9* may lead to individual differences in drug responses, resulting in variable intervention effects. Additionally, the pathogenic mechanisms of some drugs remain incompletely elucidated, limiting the development of targeted prevention and control strategies. These issues define key priorities for future research.

In summary, future studies should elucidate the association between genetic polymorphism and population heterogeneity, and develop high-risk population screening models based on genetic testing. Secondly, promote the development of novel multi-targeted and highly selective drugs to overcome the safety limitations and applicability bottlenecks of existing drugs. Furthermore, conduct large-sample evidence-based medical studies to formulate individualized prevention and treatment guidelines. Through integrated basic and clinical research, reduce the damage to patients’ multi-system health and improve the safety and rationality of clinical medication.
